# Artificial Intelligence-Driven Nanomedicine: From Drug Formulation and Nanocarrier Design to Clinical Translation

**DOI:** 10.3390/pharmaceutics18070845

**Published:** 2026-07-11

**Authors:** Abdulrahman A. Alsaqabi, Abdulaziz A. Almoutairi, Faisal Alnehari, Abdulaziz N. Alanazi, Rema Aldugiem, Yara Alsaeed, Sarah Alotaibi

**Affiliations:** Department of Pharmaceutical Sciences, College of Pharmacy, King Saud Bin Abdulaziz University for Health Sciences, Riyadh 11426, Saudi Arabia; moutiria@ksau-hs.edu.sa (A.A.A.); neharif@ksau-hs.edu.sa (F.A.); anaziabdulaz@ksau-hs.edu.sa (A.N.A.); dugiemr@ksau-hs.edu.sa (R.A.); saeedy@ksau-hs.edu.sa (Y.A.); otaibisara@ksau-hs.edu.sa (S.A.)

**Keywords:** artificial intelligence, machine learning, nanomedicine, nanocarriers, drug delivery, clinical translation

## Abstract

The integration of artificial intelligence (AI) and machine learning (ML) is fundamentally transforming pharmaceutical sciences, shifting drug formulation and nanocarrier design from traditional empirical approaches toward predictive, data-driven methodologies. By enabling the analysis of large, complex datasets, AI technologies are accelerating decision-making, improving formulation efficiency, and supporting the development of more effective therapeutic systems. Despite these advances, the successful clinical translation of advanced nanomedicines, including polymeric nanoparticles and mRNA–lipid nanoparticle platforms, remains limited by challenges such as biological barriers, highly sensitive formulation parameters, scalability issues, and the limited interpretability of many computational models. This review provides a comprehensive overview of AI applications throughout the pharmaceutical development lifecycle. It explores how classical machine learning algorithms and deep learning architectures optimize conventional dosage forms, enhance formulation development, and enable the rational design of targeted nanocarriers. Particular emphasis is placed on predicting critical quality attributes, encapsulation efficiency, physicochemical properties, drug-release behavior, therapeutic efficacy, and early-stage nanotoxicity. Furthermore, we critically assess the regulatory considerations, manufacturing constraints, data quality issues, and tumor microenvironment heterogeneity that continue to impede bench-to-clinic translation. Ultimately, overcoming these challenges requires moving beyond isolated algorithmic optimization toward an integrated framework that combines computational intelligence, robust experimental validation, and continuous clinical feedback. Such a synergistic approach is expected to drive the next generation of precision nanomedicine and facilitate the safe and effective translation of AI-enabled pharmaceutical innovations into clinical practice.

## 1. Introduction

**Figure 1:**
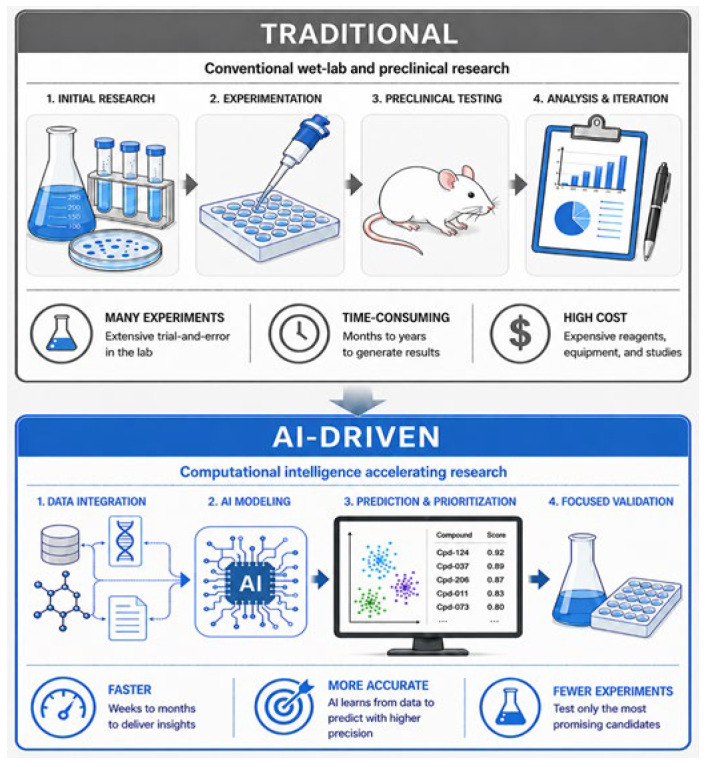
Comparison of traditional and AI-driven approaches in pharmaceutical research. Traditional methods rely on extensive, time-consuming, and costly wet-lab experimentation and trial-and-error processes. In contrast, AI-driven workflows utilize data integration and computational modeling to rapidly predict and prioritize the most promising drug candidates, thereby minimizing physical experiments, reducing costs, and accelerating the delivery of highly accurate insights.

The integration of artificial intelligence (AI) into pharmaceutical sciences has gradually reshaped how researchers approach drug discovery, formulation, and delivery. For decades, pharmaceutical development has relied on largely experimental and iterative methods that are often time-consuming, costly, and limited by an incomplete understanding of complex biological systems. Although advances in screening technologies and computational chemistry have improved efficiency, the process of translating promising drug candidates into clinically effective therapies remains slow and uncertain, with high failure rates continuing to be a major challenge in the field, as illustrated in Figure 1 [1,2]. Importantly, despite decades of methodological refinement, these translational inefficiencies persist, suggesting that the bottleneck is not purely technological but also rooted in biological complexity and the limited predictive power of existing experimental frameworks.

In this context, machine learning (ML) has become an increasingly important tool in pharmaceutical research. Rather than replacing traditional methods, ML complements them by enabling the analysis of large, complex datasets that would otherwise be difficult to interpret. In particular, ML-based approaches have been widely used to predict key absorption, distribution, metabolism, excretion, and toxicity (ADMET) properties. These predictions play an important role in early-stage drug development, where identifying safety and pharmacokinetic limitations early can significantly reduce downstream failures [1,2,3]. However, the performance of such models remains highly dependent on dataset quality and chemical space coverage, and their predictive reliability often decreases when applied to structurally novel compounds outside the training distribution. As a result, computational models are now more frequently incorporated into decision-making during lead identification and optimization, helping to prioritize compounds with more favorable profiles, although their outputs are typically interpreted as supportive rather than definitive evidence.

Beyond small-molecule drug discovery, pharmaceutical research has increasingly expanded into more complex systems, particularly nanoscale drug delivery platforms. Lipid nanoparticles, polymeric systems, nanoemulsions, and related carriers have been developed to improve solubility, stability, and targeted delivery of therapeutic agents. More recently, these systems have become central to the development of nucleic acid-based therapies, including mRNA-based drugs and vaccines, where delivery efficiency is critical to clinical success. However, despite strong preclinical performance, translating nanocarrier systems into consistent clinical outcomes remains limited, largely due to biological barriers such as immune clearance, interpatient variability, and the unpredictability of in vivo behavior. In addition, designing such systems involves many interdependent variables, making optimization through conventional experimental approaches both challenging and resource-intensive [4,5,6].

Given this complexity, interest in applying data-driven methods to formulation science has grown. Machine learning approaches can assist in understanding how formulation parameters affect performance outcomes such as particle size, encapsulation efficiency, and drug-release behavior. Rather than relying solely on trial-and-error experimentation, researchers can now use predictive models to guide formulation design and identify the most promising experimental conditions. This shift does not eliminate the need for laboratory work, but it introduces a data-dependent layer of decision-making that is still constrained by the representativeness and completeness of available datasets. These developments form the basis of Section 2, which discusses traditional formulation approaches and ADMET prediction, and Section 3, which focuses on advanced drug delivery systems and nucleic acid-based formulations.

At a more advanced level, artificial intelligence is also being explored as a tool for the rational design of nanocarriers. In this context, AI is used not only for prediction but also for optimization. By integrating diverse datasets, including physicochemical properties, biological interactions, and experimental outcomes, machine learning models can help identify design patterns that are not easily observable through conventional analysis. This includes optimizing nanocarrier size, surface characteristics, and drug-loading capacity, as well as improving stability and in vivo performance. However, these optimization frameworks often prioritize statistical performance over mechanistic interpretability, raising important questions about their robustness when applied to biologically heterogeneous systems. In some cases, multi-objective optimization approaches are used to balance competing formulation requirements, a task that is often difficult to achieve with traditional methods alone [6,7,8]. Section 4 of this review explores these developments in more detail, focusing on how AI is being used to support the design of smarter and more efficient nanocarrier systems.

Despite these promising advances, several challenges continue to limit the clinical translation of AI-driven nanomedicine. A primary limitation is the quality and consistency of available data. Many datasets used to train predictive models are incomplete, heterogeneous, or derived from different experimental conditions, which can undermine the reliability of model outputs. In addition, many advanced machine learning models are complex systems that are difficult to interpret, raising concerns about transparency and trust, especially in regulated healthcare environments. This issue is often referred to as the “black-box” problem, and it remains a key barrier to clinical adoption [8,9,10]. Efforts to improve explainability, standardize datasets, and establish robust validation frameworks are therefore becoming increasingly important, although progress in these areas remains uneven across different subfields of pharmaceutical AI.

Another important challenge is the gap between computational predictions and real-world biological systems. While models can provide useful guidance, biological complexity often introduces variability that is difficult to fully capture in silico. As a result, experimental validation remains essential, and successful translation requires close integration between computational modeling and laboratory research. Regulatory considerations further complicate this landscape, as approval pathways for AI-assisted systems are still evolving and lack full standardization in many regions, leading to uncertainty about how such tools should be evaluated within existing drug approval frameworks.

**Figure 2:**
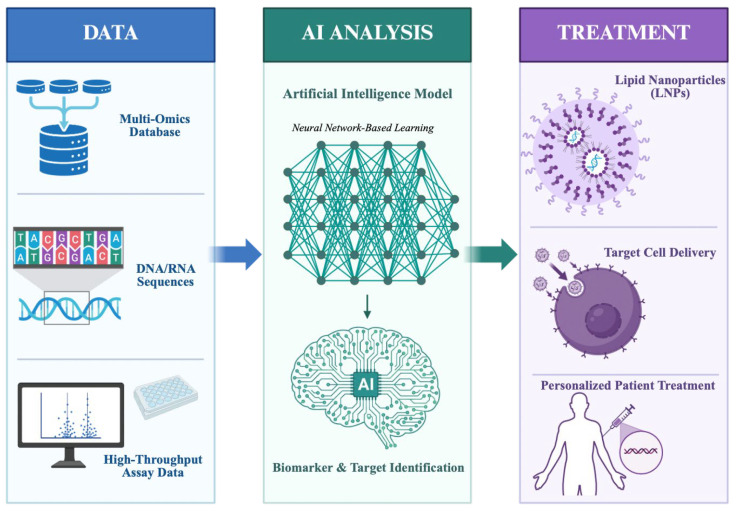
Overview of the AI-driven pharmaceutical development workflow. The process begins with the integration of complex datasets, including multi-omics databases, DNA/RNA sequences, and high-throughput assay data. These inputs are processed using advanced neural network-based AI models for biomarker and target identification, which ultimately guide the development of precision treatments such as targeted lipid nanoparticles (LNPs) and personalized patient care strategies.

In response to these challenges, this review aims to provide a structured overview of how artificial intelligence is being integrated across different stages of pharmaceutical development, as illustrated in Figure 2. It covers traditional formulation modeling and ADMET prediction, advanced nanoscale drug delivery systems, and the emerging role of AI in designing and optimizing smart nanocarriers. It also discusses key barriers to clinical translation and highlights future directions, including the potential role of digital modeling approaches in personalized nanomedicine. Section 5 focuses specifically on these translational and regulatory aspects, along with broader perspectives on the future of digital nanomedicine.

Overall, this work aims to highlight how AI is gradually becoming a practical tool in pharmaceutical sciences rather than a purely theoretical concept. While significant challenges remain, the integration of computational methods with experimental pharmaceutics is beginning to reshape how drug delivery systems are designed and evaluated. This evolving interface between data science and pharmaceutical research is likely to play an important role in developing more efficient and clinically relevant nanomedicine strategies in the coming years. Finally, AI does not eliminate uncertainty in nanomedicine; it redistributes it from the experimental space to the computational and translational layers.

This review was conducted through a comprehensive literature search of PubMed, Scopus, Web of Science, and Google Scholar databases. Relevant studies published between 2018 and 2025 were identified using combinations of keywords including “artificial intelligence”, “machine learning”, “drug formulation”, “ADMET prediction”, “nanomedicine”, “nanocarriers”, “lipid nanoparticles”, “mRNA delivery”, and “clinical translation”. Original research articles, reviews, and regulatory reports written in English were considered. Studies were selected based on their relevance to AI-driven pharmaceutical formulation, nanocarrier optimization, toxicity prediction, and clinical translation. Additional references were identified through manual screening of reference lists from key publications.

## 2. Machine Learning Algorithms in Traditional Pharmaceutical Formulation and ADMET Property Prediction

The conventional development of pharmaceutical formulations has long relied on empirical, trial-and-error approaches that are both time-consuming and resource-intensive, particularly when dealing with structurally complex drug candidates [11]. In recent years, machine learning (ML) has emerged as a practical alternative for extracting predictive relationships from existing experimental datasets. However, the field continues to face important limitations, including variable data quality, limited interpretability of model outputs, and challenges in transferring predictions across chemically diverse domains [11]. Importantly, these limitations are not merely technical constraints but represent fundamental bottlenecks that directly affect the reliability and real-world applicability of ML models in pharmaceutics. Although many models demonstrate high predictive performance under controlled experimental conditions, this success often fails to translate to practical formulation development. One major reason is historical dataset bias, as the published literature and publicly available databases are heavily enriched with successful (“positive”) outcomes while failed formulations or inactive compounds (“negative” data) remain underreported. Because negative examples are essential for defining robust decision boundaries, this imbalance limits model generalizability. In addition, the lack of standardized data generation protocols across laboratories introduces substantial experimental heterogeneity. Differences in raw material sourcing, equipment calibration, and environmental conditions generate batch-to-batch variability that becomes embedded within aggregated datasets. Consequently, ML models trained on such heterogeneous and biased data may inadvertently learn experimental artifacts rather than true formulation–property relationships, thereby reducing their predictive reliability when applied to novel pharmaceutical systems [12].

Among the earliest and most widely adopted approaches, artificial neural networks (ANNs) have played a central role in modeling non-linear relationships between formulation variables and product performance. By approximating complex input–output mappings, these models can significantly reduce the need for extensive experimental screening, effectively replacing large numbers of laboratory iterations with computational predictions [13]. However, despite their apparent efficiency, artificial neural networks often function as data-interpolation tools rather than true mechanistic models. This distinction is critical because it limits their ability to extrapolate beyond the chemical space represented in the training data, raising concerns about their reliability when applied to novel drug candidates.

In solid dosage form development, predictive modeling must account for multiple interacting variables, including critical material attributes and associated pharmacokinetic behavior [14]. This requires careful dataset preparation, where issues such as class imbalance and high dimensionality are commonly addressed through preprocessing strategies including oversampling and feature reduction techniques [14]. In this context, classical ML approaches, such as Support Vector Machines (SVM) and multivariate statistical models, have demonstrated clear value in improving formulation efficiency and robustness [15]. More advanced ensemble learning methods, including Random Forests and Voting Regressors, have also been applied to predict key in vitro quality attributes, such as disintegration time, friability, and water uptake, thereby reducing experimental workload and material waste [16]. Similarly, gradient-based tree models, such as Gradient Boosting and Extra Trees, have shown strong performance in predicting solubility in complex solvent systems where traditional thermodynamic approaches are often insufficient [17]. Nevertheless, the apparent improvements in predictive accuracy reported for ensemble methods may be partially influenced by dataset-specific optimization rather than true generalizable learning. As a result, performance metrics often vary significantly across independent validation studies, highlighting the need for more rigorous external validation frameworks.

Beyond formulation science, ML has become increasingly important in early-stage drug discovery, particularly for predicting ADMET properties. These models integrate diverse data types, including molecular fingerprints, SMILES representations, protein sequences, and graph-based biological descriptors [18]. This multimodal integration enables a more holistic representation of drug–target interactions and supports more reliable prediction of pharmacokinetic and toxicity profiles. Recent advances in deep learning have further expanded these capabilities, particularly through models that infer the structural and functional properties of proteins from sequence data [19]. These structural insights have enabled more realistic virtual screening workflows, in which protein–ligand complexes are encoded as high-dimensional representations suitable for convolutional neural network analysis [20]. In parallel, generative models combined with reinforcement learning have driven a shift toward de novo drug design, enabling exploration of large chemical spaces and the identification of novel compounds with desirable biological activity and synthetic feasibility [21]. A notable example of this paradigm is the discovery of halicin, a structurally novel antibacterial compound identified through deep learning-based screening of large chemical libraries [22]. This demonstrates the ability of ML systems to move beyond human-designed chemical intuition and uncover unexpected therapeutic candidates.

To illustrate the practical utility of these computational models, several recent studies have successfully integrated AI predictions with rigorous experimental validation. In the context of formulation optimization, for instance, machine learning algorithms such as Random Forests and Artificial Neural Networks have been explicitly deployed to optimize the composition of amorphous solid dispersions (ASDs). By predicting the physical stability and dissolution rates of various drug–polymer combinations, researchers were able to synthesize the top-ranked AI-designed formulations, which subsequently demonstrated in vitro dissolution profiles that closely matched the in silico predictions [23]. Similarly, in ADMET profiling, hybrid deep learning models have been utilized to predict the hepatotoxicity and metabolic clearance of novel small-molecule candidates. In a notable case study, an AI-driven platform screened a library of compounds for metabolic stability; the top-tier candidates were synthesized and evaluated using human liver microsome (HLM) assays, revealing a high concordance between predicted systemic clearance rates and experimental in vitro data [24]. These case studies underscore that while AI significantly narrows the experimental search space, empirical validation remains indispensable for confirming both formulation robustness and safety profiles.

A comparative overview of classical machine learning and deep learning architectures, alongside their specific pharmaceutical applications, is summarized in Table 1.

Interestingly, the applicability of these algorithms extends beyond pharmaceutical systems. Tree-based models such as Random Forests have also been successfully applied in unrelated engineering contexts, including the prediction of mechanical properties of granular materials [25]. This cross-domain success highlights the general strength of these methods for handling complex, non-linear relationships, which are also characteristic of pharmaceutical powder behavior and formulation processes. Despite these advances, the long-term reliability of ML models in pharmaceutics strongly depends on maintaining model validity over time through active lifecycle management. As chemical spaces continuously evolve and novel therapeutic targets emerge, static models inherently suffer from performance degradation, a phenomenon known as “concept drift”. Studies evaluating production-scale ADMET models have shown that while some predictions remain stable, error rates for structurally novel compounds increase over time, emphasizing the critical need for continuous model retraining pipelines that systematically incorporate newly generated experimental data [26]. Furthermore, internal cross-validation alone is insufficient to guarantee real-world robustness. To ensure that algorithms do not merely memorize dataset-specific artifacts (overfitting), rigorous external validation is mandatory. This involves testing the updated models against completely independent, temporally separated, or multi-institutional datasets, thereby confirming their true generalizability before deployment in early-stage drug discovery [27].

While machine learning methods such as XGBoost and GLMnet have demonstrated strong performance in predicting key pharmacokinetic parameters like systemic clearance and area under the curve (AUC), these purely data-driven models often lack the mechanistic interpretability provided by traditional pharmacometric approaches. To bridge this gap, there is a growing shift toward hybrid modeling frameworks that integrate machine learning with Physiologically Based Pharmacokinetic (PBPK) models. Traditional PBPK models are highly mechanistic, relying on known anatomical and physiological parameters to simulate drug disposition across distinct body compartments; however, they often struggle when faced with incomplete specific physicochemical data or complex metabolic pathways. By synergizing ML with PBPK modeling, AI can accurately predict unknown drug-specific input parameters (such as partition coefficients or intrinsic clearance) from molecular structures, which are subsequently fed into the PBPK framework. This hybrid AI/ML-PBPK approach not only preserves the mechanistic transparency required by regulatory agencies but also significantly enhances predictive accuracy across diverse patient populations, ultimately accelerating precision dosing and early-stage lead optimization.

To improve data reliability and workflow reproducibility, automated platforms such as KNIME are widely used for data mining, feature selection, and consensus modeling [28]. These tools help reduce noise in experimental datasets and improve the robustness of downstream predictions. In pharmacokinetic modeling, ML methods such as XGBoost and GLMnet have demonstrated strong performance in predicting key parameters, including drug clearance and area under the curve (AUC) [29]. However, despite their predictive accuracy, these methods often lack the mechanistic interpretability of traditional pharmacometric approaches, such as non-linear mixed-effects (NLME) modeling. As a result, hybrid frameworks that combine ML-based prediction with mechanistic PK/PD modeling are increasingly being explored to balance accuracy with biological interpretability [30]. Compelling case studies demonstrating this synergy have been recently detailed, showcasing the direct integration of machine learning within physiologically based pharmacokinetic (PBPK) frameworks. For instance, researchers have successfully utilized machine learning algorithms to accurately predict complex chemical-specific parameters—such as tissue-to-plasma partition coefficients and intrinsic clearance—based directly on molecular descriptors. Rather than functioning as standalone predictive tools, these ML-derived parameters were systematically embedded into mechanistic PBPK models to accurately simulate in vivo concentration–time profiles and human exposure levels. This hybrid ML-PBPK modeling approach significantly outperforms traditional empirical allometric scaling methods, effectively bridging the gap between data-driven predictive accuracy and biological interpretability for reliable translational dosing [31].

A major challenge across all ML applications in pharmaceutics remains the lack of standardized evaluation frameworks and consistent validation practices. Benchmarking platforms such as MoleculeNet have therefore become essential, providing standardized datasets (e.g., Tox21 and ClinTox) and evaluation protocols, such as scaffold splitting, to assess generalization to novel chemical structures [32]. When artificial intelligence models are tasked with predicting the properties of structurally novel compounds that fall outside their training distribution, their predictive performance frequently degrades. This highlights a fundamental limitation in structural extrapolation, where models may confidently output erroneous predictions for unfamiliar chemotypes. To mitigate this risk, modern computational workflows increasingly incorporate Uncertainty Quantification (UQ) and rigorously define the model’s Applicability Domain (AD)—the chemical space within which predictions can be trusted. Consequently, benchmarking platforms such as MoleculeNet have become essential, providing standardized datasets and evaluation protocols, most notably scaffold splitting. Unlike conventional random data splitting, scaffold splitting forces the model to evaluate distinct chemical backbones not present in the training set, thereby offering a highly realistic estimation of how the algorithm will perform on truly novel drug candidates during early-stage discovery [32,33].

Overall, although machine learning methods have substantially advanced pharmaceutical modeling, their current role remains largely supportive rather than transformative. The field continues to face a structural limitation: most models are trained on historically biased datasets that reflect narrow experimental conditions rather than the full diversity of clinically relevant chemical space. Without systematic improvements in data curation, external validation, and mechanistic integration, the predictive utility of these models will remain constrained within predefined domains of applicability.

## 3. Advanced Nanoscale Drug Delivery Systems and mRNA and Vaccine Formulation

The development of nanoscale drug delivery systems has transformed pharmaceutical science by enabling more controlled and targeted therapeutic delivery [34,35]. However, nanocarrier performance remains highly dependent on particle composition, surface properties, and disease context, limiting the establishment of universal design principles.

Polymeric nanoparticles are among the most established nanocarriers, offering sustained drug release through gradual polymer degradation [36,37]. PLGA is particularly valued for its biocompatibility, biodegradability, and versatility in systemic and localized delivery, including transdermal applications [38]. Despite these advantages, sustained release does not necessarily improve cellular uptake or tissue specificity, limiting clinical translation.

Lipid-based systems, particularly liposomes, have also become key drug delivery platforms due to their ability to encapsulate both hydrophilic and hydrophobic agents [39]. They are especially useful for gene delivery by protecting nucleic acids from enzymatic degradation [40]. Although several liposomal formulations have reached clinical use, their broader translation remains limited by instability during circulation and inconsistent tumor accumulation [41]. Surface modification with targeting ligands has improved selectivity but has not fully overcome these challenges [42].

The development of lipid nanoparticles (LNPs) marked a major advance in nucleic acid delivery by improving intracellular transport [43]. Typical LNPs contain ionizable lipids, cholesterol, helper phospholipids, and PEGylated lipids, which collectively determine stability, encapsulation efficiency, and in vivo performance [44]. However, slight changes in formulation can substantially alter biodistribution, immune activation, and therapeutic efficacy, complicating large-scale manufacturing.

The clinical importance of LNPs became evident with mRNA-based COVID-19 vaccines, whose rapid development demonstrated the effectiveness of mRNA-loaded LNPs [45]. Nevertheless, this success occurred under unique regulatory and clinical conditions that are not representative of oncology or chronic diseases, where long-term safety and biodistribution remain major challenges [46]. Consequently, LNPs have become the leading non-viral mRNA delivery platform, supported by continued innovation in lipid composition and formulation strategies [47,48,49,50].

Current research aims to improve delivery efficiency while reducing toxicity and off-target effects through optimization of lipid composition and helper lipids [51,52]. However, enhancing one property often compromises others, such as immunogenicity or biodistribution, highlighting the need to balance multiple formulation objectives. These advances have also expanded LNP delivery beyond the liver to extrahepatic tissues [53].

Beyond infectious diseases, mRNA-LNPs are increasingly investigated for cancer therapy by encoding tumor-associated antigens and immunomodulatory proteins to stimulate targeted immune responses [54]. This strategy enables transient therapeutic protein production within patient cells and supports personalized treatment approaches [55]. However, tumor heterogeneity, immune evasion, and variability of the tumor microenvironment continue to limit clinical translation.

Naturally derived nanocarriers, particularly exosomes, have also attracted attention because of their intrinsic biocompatibility and ability to cross biological barriers. Despite their promise, clinical application remains limited by challenges in large-scale production, purification, reproducibility, heterogeneous cargo loading, and low manufacturing yield [56].

To consolidate the physicochemical principles discussed, Table 2 summarizes the key properties, major advantages, current limitations, and clinical translation status of the primary nanocarrier platforms.

## 4. Integrating Artificial Intelligence and Machine Learning in Optimizing and Designing Smart Nanocarriers

The application of machine learning in drug delivery has gradually shifted the field from largely empirical optimization to a more predictive and data-driven paradigm [57]. In nanomedicine, this transition is particularly significant due to the inherently high-dimensional nature of formulation design, in which multiple interdependent physicochemical and biological variables must be optimized simultaneously [58]. Although machine learning tools have demonstrated clear value in accelerating formulation screening and narrowing experimental search spaces [59], their impact remains strongly dependent on the quality and representativeness of underlying datasets, which are often limited by experimental bias and incomplete biological characterization. As a result, the predictive gains observed in silico do not always translate directly into reproducible in vivo performance [60].

One of the most prominent applications of these methods lies in the rational design of targeted nanocarriers. Machine learning models can assist in optimizing surface functionalization and ligand–receptor interactions, both of which are critical determinants of cellular uptake and tissue specificity [61]. However, these predictions are typically derived under simplified biological assumptions, which may not fully capture the dynamic and heterogeneous nature of physiological environments. Consequently, AI-guided design is better interpreted as a prioritization tool rather than a definitive optimization framework, supporting experimental decision-making rather than replacing it.

In addition, predictive models have been employed to estimate formulation behavior and drug-release characteristics, particularly in long-acting polymer-based delivery systems, where release kinetics are a key design constraint [62]. Beyond formulation-level predictions, some models extend to forecasting in vivo performance, including anticancer efficacy, based on structural and physicochemical descriptors [63]. Nevertheless, such extrapolations remain inherently uncertain, as they rely on indirect correlations rather than a mechanistic understanding of biological interactions, limiting their robustness outside well-characterized chemical spaces.

A particularly active area of development has been lipid nanoparticle (LNP) design for mRNA delivery. Machine learning approaches are increasingly used to navigate the vast and non-linear formulation space of LNP systems, identifying compositions associated with improved stability and delivery efficiency [64]. While these approaches have contributed to significant formulation advances, the LNP design landscape remains highly sensitive, where small perturbations in lipid composition can lead to disproportionately large changes in biological outcomes. This sensitivity highlights a persistent limitation of data-driven optimization: the inability to fully capture nonlinear physiological feedback mechanisms governing intracellular delivery and immune recognition [65]. Because improving one functional aspect of LNPs—such as maximizing mRNA encapsulation efficiency—often inadvertently compromises another, such as increasing particle size or inducing higher systemic toxicity, single-objective algorithms are generally insufficient. To systematically address these competing formulation requirements, multi-objective optimization (MOO) strategies, such as Pareto frontier analysis and genetic algorithms, are increasingly being integrated into LNP design workflows. These advanced computational approaches allow researchers to simultaneously co-optimize conflicting parameters, identifying the optimal molar ratios of ionizable lipids, cholesterol, and PEG–lipids that balance maximum transfection efficacy with minimal cytotoxicity and acceptable biodistribution profiles. This holistic MOO framework is crucial for efficiently navigating the vast combinatorial lipid space and identifying clinically viable LNP candidates that would be easily missed by traditional sequential experimentation.

To bridge the gap between theoretical models and practical application, the recent literature provides compelling case studies where AI-guided nanocarrier optimization was explicitly validated through experimental synthesis and biological testing. Within the domain of polymeric nanocarriers, ensemble machine learning algorithms such as Random Forest and artificial neural networks (ANNs) have been actively deployed to optimize the critical quality attributes of drug-loaded PLGA nanoparticles. These computational models accurately predicted the non-linear relationships between polymer concentrations, surfactant ratios, and formulation parameters, allowing researchers to precisely tune particle size and maximize encapsulation efficiency prior to physical synthesis. Subsequent laboratory validations confirmed that these AI-optimized PLGA nanoparticles matched the in silico predictions with exceptional accuracy [66]. Similarly, in the development of lipid-based platforms, researchers have recently utilized machine learning frameworks to systematically optimize the composition of lipid nanoparticles (LNPs) for nucleic acid delivery. By applying deep learning algorithms to screen combinatorial lipid libraries, predictive models successfully identified optimized LNP formulations that bypassed traditional trial-and-error bottlenecks. When these AI-prioritized LNPs were synthesized and evaluated in vivo, they demonstrated significantly enhanced systemic stability, targeted cellular uptake, and improved transfection efficiency compared to empirically designed baselines [67]. These case studies conclusively demonstrate that integrating machine learning with targeted experimental validation can successfully transition nanocarrier design from a predominantly empirical process toward a robust, predictive engineering pipeline.

Beyond efficacy, physicochemical stability and safety remain central challenges in nanocarrier engineering. Machine learning models have been used to estimate key parameters such as zeta potential, which is closely associated with colloidal stability in biological fluids [68]. Similarly, drug-loading capacity and encapsulation efficiency—long-standing bottlenecks in nanomedicine development—can now be partially optimized using predictive modeling approaches [69]. However, these predictions are often constrained by narrow training distributions and may not generalize well across different nanocarrier architectures or manufacturing scales. Extensions of these methods to systems such as liposomes and niosomes further demonstrate their versatility, but also expose variability in predictive reliability across formulation classes [70].

Importantly, machine learning is increasingly being explored for early-stage safety assessment, particularly in silico prediction of nanomaterial cytotoxicity [71]. This represents a valuable step toward reducing experimental burden and improving screening efficiency. However, toxicity prediction remains one of the most uncertain areas of nanomedicine modeling due to the complexity of biological response pathways and the limited availability of high-quality, standardized toxicity datasets. This limitation is especially pronounced in inorganic nanomaterials and emerging cell-based delivery platforms, where fundamentally different factors drive data scarcity. For inorganic nanomaterials, such as metallic or mesoporous silica nanoparticles, data scarcity and inconsistency stem from the vast parametric space of their synthesis—where minute variations in size, geometry, and surface capping drastically alter biological responses. Furthermore, many metallic nanoparticles optically interfere with standard colorimetric toxicity assays, leading to irreproducible reporting across laboratories. Conversely, for cell-based delivery systems like exosomes or cell-membrane-coated nanocarriers, the data bottleneck is driven by profound biological heterogeneity. The inherent donor-to-donor variability, dynamic molecular cargo (comprising complex mixtures of proteins, lipids, and nucleic acids), and the lack of universally standardized isolation protocols make it exceedingly difficult to construct the large, highly curated, and reproducible datasets required to effectively train robust deep learning algorithms [72,73].

Overall, while machine learning has become an indispensable tool in nanocarrier design and optimization, its current role is primarily supportive rather than autonomous. The field still faces a fundamental limitation: most predictive models operate within constrained chemical and biological domains that do not fully capture in vivo complexity. Without deeper integration of mechanistic understanding and standardized biological datasets, AI-driven nanocarrier design will remain a powerful but inherently bounded approach rather than a fully predictive engineering framework. The comprehensive role of artificial intelligence across the entire nanomedicine workflow, from initial design to safety prediction and clinical translation, is outlined in Table 3.

## 5. Clinical Translation, Regulatory Hurdles, and Future Perspectives of Digital Nanomedicine

Despite major advances in nanocarrier design and computational optimization, clinical translation remains a complex and non-linear process [74]. While machine learning and digital health have improved patient stratification, treatment monitoring, and trial optimization [75], they have not fully bridged the gap between preclinical success and clinical efficacy, as reflected by the slow and unpredictable progression of many anticancer nanomedicines through clinical trials [76].

Nanomedicine also presents regulatory challenges because of the dynamic behavior of nanoparticles in biological systems. Regulatory agencies have adopted more specialized, risk-based frameworks to address nanoscale-specific pharmacokinetics and safety [77]. However, the lack of standardized experimental protocols and clinical reporting continues to hinder cross-study comparison and the development of unified regulatory pathways.

A major obstacle to clinical translation is the unpredictable in vivo behavior of nanomaterials. Protein corona formation and rapid clearance by the mononuclear phagocyte system can substantially alter nanoparticle biodistribution and therapeutic efficacy [78]. These effects vary across biological systems, limiting the predictive value of animal models. In oncology, tumor microenvironment heterogeneity further complicates drug accumulation and treatment response [79].

The variability of the enhanced permeability and retention (EPR) effect in human tumors has substantially reduced the reliability of passive targeting strategies [80]. To address this unpredictability, artificial intelligence (AI) is increasingly being leveraged to non-invasively assess and predict patient-specific EPR characteristics. By applying machine learning algorithms to routine medical imaging modalities, such as dynamic contrast-enhanced MRI and PET, researchers can extract high-dimensional radiomic features that correlate with tumor vascular permeability, interstitial fluid pressure, and extracellular matrix density. This AI-driven radiomic approach enables pre-treatment patient stratification by identifying individuals with favorable EPR profiles who are most likely to benefit from passive nanocarrier accumulation. As a result, AI-assisted prediction has the potential to improve patient selection and reduce the clinical attrition historically associated with empiric administration of passive nanomedicines [81]. Nevertheless, because passive targeting remains inherently dependent on highly heterogeneous tumor biology, research has increasingly shifted toward actively targeted and stimuli-responsive delivery systems. While these strategies offer greater targeting specificity, they also introduce additional challenges related to reproducibility, large-scale manufacturing, and regulatory approval. Consequently, modern nanomedicine development has evolved beyond maximizing therapeutic efficacy alone, placing equal emphasis on manufacturability, robustness, and clinically relevant outcomes to facilitate successful clinical translation [82,83].

Future progress is expected to rely on adaptive and intelligent nanomedicine platforms with improved spatial and temporal control of drug release [84]. However, experience from both successful and unsuccessful clinical trials demonstrates that technological advances alone are insufficient for clinical translation [85]. Instead, success depends on integrating biological understanding with scalable manufacturing and regulatory feasibility while maintaining cost-effectiveness and reproducibility [86,87].

To achieve this scalable and compliant framework, the integration of Artificial Intelligence with Quality by Design (QbD) principles is becoming indispensable. AI accelerates the QbD paradigm by rapidly mapping complex multidimensional formulation spaces, allowing developers to identify critical process parameters without exhaustive trial-and-error. Furthermore, the advent of “digital twins”—virtual, real-time computational replicas of physical manufacturing processes—offers a transformative approach to clinical scale-up. By simulating physical stress, fluid dynamics, and thermodynamic variations in silico, digital twins enable continuous real-time monitoring and predictive maintenance, drastically reducing batch failures during the mass production of sensitive nanocarriers like LNPs. However, maximizing the impact of these digital innovations requires urgent regulatory harmonization. As regulatory bodies grapple with the dynamic nature of both nanomedicines and machine learning algorithms, establishing unified, cross-regional guidelines for the validation of AI-generated data, digital twin simulations, and continuous manufacturing processes is essential to streamline global approval pathways and ensure consistent patient safety [88].

**Figure 3:**
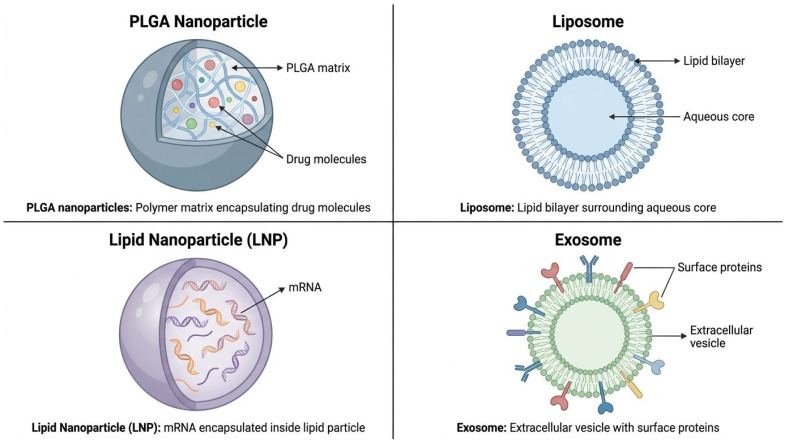
Overview of common nanocarriers for drug delivery. The schematic illustrates four primary platforms: PLGA nanoparticles, which use a degradable polymer matrix to encapsulate drug molecules; liposomes, characterized by a lipid bilayer surrounding an aqueous core; lipid nanoparticles (LNPs), specifically designed to encapsulate and protect mRNA payloads; and exosomes, which are natural extracellular vesicles equipped with surface proteins that facilitate cellular targeting.

Ultimately, the integration of nanotechnology, data science, and clinical medicine is expected to advance precision nanomedicine [89]. Rather than replacing experimental research, computational tools are likely to function within hybrid frameworks where predictive modeling, experimental validation, and clinical feedback continuously refine therapeutic development. An overview of common nanocarriers, including PLGA nanoparticles, liposomes, lipid nanoparticles (LNPs), and exosomes, is presented in Figure 3.

## Figures and Tables

**Table 1:** Comparative Overview of Classical Machine Learning and Deep Learning Architectures and Their Pharmaceutical Applications.

Algorithm Category	Specific Algorithms	Key Inputs	Key Outputs	Pharmaceutical Applications
Classical Machine Learning	Random Forest (RF), eXtreme Gradient Boosting (XGBoost)	Process parameters, physicochemical descriptors, SMILES strings	Disintegration time, solubility, systemic clearance, AUC	Solid dosage formulation, early-stage PK/PD prediction, material behavior modeling
Classical Machine Learning	Support Vector Machines (SVM), Multivariate Data Analysis	Formulation composition, material attributes	Stability, tablet friability, water absorption ratios	Quality control, scale-up optimization, material waste reduction
Deep Learning	Artificial Neural Networks (ANNs)	Non-linear process variables, complex biological assay data	Optimized excipient ratios, ADMET properties	High-throughput virtual screening, non-linear formulation optimization
Deep Learning	Graph Neural Networks (GNNs)	Molecular graphs (interconnected nodes and edges)	Target affinity, bioactivity, complex representations	De novo drug design, advanced molecular property prediction
Deep Learning	Convolutional Neural Networks (CNNs)	3D protein structures, interaction fingerprints	Binding poses, virtual screening prioritization	Target-ligand interaction modeling, structural bioinformatics

**Table 2:** Key properties, major advantages, current limitations, and clinical translation status of the primary nanocarrier platforms.

Nanocarrier Platform	Key Physicochemical Properties	Major Advantages	Current Limitations	Clinical Translation Status
Polymeric Nanoparticles (e.g., PLGA)	Tunable degradation rates, variable size and surface charge	Sustained and controlled drug release, high biocompatibility, versatile payload	Potential acidic microenvironments upon degradation, complex scale-up	Highly established (Multiple FDA-approved formulations)
Liposomes	Phospholipid bilayer structure, aqueous core, amphiphilic nature	High biocompatibility, low toxicity, encapsulates both hydrophilic and hydrophobic drugs	Short in vivo half-life (if unPEGylated), susceptible to oxidation and rapid clearance	Mature and widely translated (e.g., Doxil, Onivyde)
Lipid Nanoparticles (LNPs)	Solid lipid core, incorporates ionizable cationic lipids and PEG	Robust nucleic acid encapsulation, facilitates efficient endosomal escape	Potential immunogenicity, complex intellectual property landscape, off-target liver accumulation	Highly advanced (Gold standard for mRNA COVID-19 vaccines)
Exosomes (Extracellular Vesicles)	Endogenous lipid bilayer, cell-derived surface proteins	Exceptional biocompatibility, inherent ability to cross formidable biological barriers	Complex isolation and purification processes, low yield, high batch-to-batch variability	Emerging (Increasingly evaluated in early clinical trials)

**Table 3:** Explains the comprehensive role of artificial intelligence across the entire nanomedicine workflow, from initial design to safety prediction and clinical translation.

Development Phase	Role of Artificial Intelligence	Key Functions & Outputs	Representative Examples
Design & Formulation	Predicting optimal nanocarrier composition and component compatibility	Excipient selection, entrapment efficiency prediction, particle size and stability optimization	Optimizing curcumin-loaded liposomes; identifying highly stable mRNA-LNP lipid ratios
Targeting & Optimization	Enhancing spatial precision and target site accumulation	Modeling ligand-receptor binding kinetics, optimizing surface functionalization	Predicting anti-cancer efficacy and cellular uptake based on specific structural modifications
Safety & ADMET Profiling	Early-stage screening of toxicological and pharmacokinetic profiles	Estimating zeta potential, in silico cellular toxicity predictions, systemic clearance rates	Quantitative prediction of inorganic nanomaterial cytotoxicity prior to physical synthesis
Clinical Translation & Manufacturing	Facilitating process scale-up and personalized therapeutic delivery	Establishing digital twins, Quality by Design (QbD) compliance, patient cohort stratification	Adapting real-time quality control for precision nanotherapeutics

## Data Availability

No new data was created or analyzed in this work. Data sharing is not applicable in this article.

## References

[1] Ekins S., Puhl A.C., Zorn K.M., Lane T.R., Russo D.P., Klein J.J., Hickey A.J., Clark A.M. (2019). Exploiting machine learning for end-to-end drug discovery and development. Nat. Mater..

[2] Vamathevan J., Clark D., Czodrowski P., Dunham I., Ferran E., Lee G., Li B., Madabhushi A., Shah P., Spitzer M. (2019). Applications of machine learning in drug discovery and development. Nat. Rev. Drug Discov..

[3] Paul D., Sanap G., Shenoy S., Kalyane D., Kalia K., Tekade R.K. (2021). Artificial intelligence in drug discovery and development. Drug Discov. Today.

[4] Schneider P., Walters W.P., Plowright A.T., Sieroka N., Listgarten J., Goodnow R.A., Fisher J., Jansen J.M., Duca J.S., Rush T.S. (2020). Rethinking drug design in the artificial intelligence era. Nat. Rev. Drug Discov..

[5] Suriyaamporn P., Pamornpathomkul B., Patrojanasophon P., Ngawhirunpat T., Rojanarata T., Opanasopit P. (2024). The Artificial Intelligence-Powered New Era in Pharmaceutical Research and Development: A Review. AAPS PharmSciTech.

[6] Kim J., Park S., Min D., Kim W. (2021). Comprehensive Survey of Recent Drug Discovery Using Deep Learning. Int. J. Mol. Sci..

[7] Li H., Zou L., Kowah J.A.H., He D., Liu Z., Ding X., Wen H., Wang L., Yuan M., Liu X. (2023). A compact review of progress and prospects of deep learning in drug discovery. J. Mol. Model..

[8] Raza A., Chohan T.A., Buabeid M., Arafa E.S.A., Chohan T.A., Fatima B., Murtaza G. (2023). Deep learning in drug discovery: A futuristic modality to materialize the large datasets for cheminformatics. J. Biomol. Struct. Dyn..

[9] Askr H., Elgeldawi E., Aboul Ella H., Elshaier Y.A.M.M., Gomaa M.M., Hassanien A.E. (2023). Deep learning in drug discovery: An integrative review and future challenges. Artif. Intell. Rev..

[10] Murray J.D., Lange J.J., Bennett-Lenane H., Holm R., Kuentz M., O’Dwyer P.J., Griffin B.T. (2023). Advancing algorithmic drug product development: Recommendations for machine learning approaches in drug formulation. Eur. J. Pharm. Sci..

[11] Wang S., Di J., Wang D., Dai X., Hua Y., Gao X., Zheng A., Gao J. (2022). State-of-the-Art Review of Artificial Neural Networks to Predict, Characterize and Optimize Pharmaceutical Formulation. Pharmaceutics.

[12] Bender A., Cortés-Ciriano I. (2021). Artificial intelligence in drug discovery: What is realistic, what are illusions? Part 1: Ways to make an impact, and why we are not there yet. Drug Discov. Today.

[13] Jiang J., Ma X., Ouyang D., Williams R.O. (2022). Emerging Artificial Intelligence (AI) Technologies Used in the Development of Solid Dosage Forms. Pharmaceutics.

[14] Sahu A., Rathee S., Saraf S., Jain S.K. (2024). A Review on the Recent Advancements and Artificial Intelligence in Tablet Technology. Curr. Drug Targets.

[15] Momeni M., Afkanpour M., Rakhshani S., Mehrabian A., Tabesh H. (2024). Advancing pharmaceutical Intelligence via computationally Prognosticating the in-vitro parameters of fast disintegration tablets using Machine Learning models. BMC Med. Inform. Decis. Mak..

[16] Ghazwani M., Begum M.Y. (2023). Computational intelligence modeling of hyoscine drug solubility and solvent density in supercritical processing: Gradient boosting, extra trees, and random forest models. Sci. Rep..

[17] Shi W., Yang H., Xie L., Yin X.X., Zhang Y. (2024). A review of machine learning-based methods for predicting drug-target interactions. Health Inf. Sci. Syst..

[18] Senior A.W., Evans R., Jumper J., Kirkpatrick J., Sifre L., Green T., Qin C., Žídek A., Nelson A.W.R., Bridgland A. (2020). Improved protein structure prediction using potentials from deep learning. Nature.

[19] Kimber T.B., Chen Y., Volkamer A. (2021). Deep learning in virtual screening: Recent applications and developments. Int. J. Mol. Sci..

[20] Zhavoronkov A., Ivanenkov Y.A., Aliper A., Veselov M.S., Aladinskiy V.A., Aladinskaya A.V., Terentiev V.A., Polykovskiy D.A., Kuznetsov M.D., Asadulaev A. (2019). Deep learning enables rapid identification of potent DDR1 kinase inhibitors. Nat. Biotechnol..

[21] Stokes J.M., Yang K., Swanson K., Jin W., Cubillos-Ruiz A., Donghia N.M., MacNair C.R., French S., Carfrae L.A., Bloom-Ackermann Z. (2020). A Deep Learning Approach to Antibiotic Discovery. Cell.

[22] Zhou J., Li E., Wei H., Li C., Qiao Q., Armaghani D.J. (2019). Random Forests and Cubist Algorithms for Predicting Shear Strengths of Rockfill Materials. Appl. Sci..

[23] Han R., Xiong H., Ye Z., Yang Y., Huang T., Jing Q., Ouyang D. (2023). The applications of machine learning to predict the forming of chemically stable amorphous solid dispersions prepared by hot-melt extrusion. Int. J. Pharm. X.

[24] Sakiyama Y., Yuki H., Moriya T., Hattori K., Suzuki M., Shimada K., Honma T. (2008). Predicting human liver microsomal stability with machine learning techniques. J. Mol. Graph Model..

[25] Sheridan R.P. (2022). Stability of Prediction in Production ADMET Models as a Function of Version: Why and When Predictions Change. J. Chem. Inf. Model..

[26] Falcón-Cano G., Molina C., Cabrera-Pérez M.Á. (2023). ADMET prediction with deep learning: State of the art and future perspectives. Adv. Drug Deliv. Rev..

[27] Chicco D., Jurman G. (2022). The ABC recommendations for validation of supervised machine learning results in biomedical sciences. Front. Big Data.

[28] Ahmadi M., Alizadeh B., Ayyoubzadeh S.M., Abiyarghamsari M. (2024). Predicting Pharmacokinetics of Drugs Using Artificial Intelligence Tools: A Systematic Review. Eur. J. Drug Metab. Pharmacokinet..

[29] Smajić A., Keilhofer J., Bieri M., Hemmerich J. (2023). Machine learning for the prediction of pharmacokinetic parameters: A review. Expert Opin. Drug Metab. Toxicol..

[30] Wu Z., Ramsundar B., Feinberg E.N., Gomes J., Geniesse C., Pappu A.S., Leswing K., Pande V. (2017). MoleculeNet: A benchmark for molecular machine learning. Chem. Sci..

[31] Chou W.C., Lin Z. (2023). Machine learning and artificial intelligence in physiologically based pharmacokinetic modeling. Toxicol. Sci..

[32] Wieder O., Kohlbacher S., Mélançon M., Seidel T., Langer T. (2020). A compact review of molecular property prediction with graph neural networks. Drug Discov. Today.

[33] Scalia G., Grambow C.A., Pernici B., Li Y.P., Green W.H. (2020). Evaluating Scalable Uncertainty Estimation Methods for Deep Learning-Based Molecular Property Prediction. J. Chem. Inf. Model..

[34] Mitchell M.J., Billingsley M.M., Haley R.M., Wechsler M.E., Peppas N.A., Langer R. (2021). Engineering precision nanoparticles for drug delivery. Nat. Rev. Drug Discov..

[35] Blanco E., Shen H., Ferrari M. (2015). Principles of nanoparticle design for overcoming biological barriers to drug delivery. Nat. Biotechnol..

[36] Kamaly N., Yameen B., Wu J., Farokhzad O.C. (2016). Degradable Controlled-Release Polymers and Polymeric Nanoparticles: Mechanisms of Controlling Drug Release. Chem. Rev..

[37] Kumar L., Kukreti G., Rana R., Chaurasia H., Sharma A., Sharma N., Komal (2023). Poly(lactic-co-glycolic) Acid (PLGA) Nanoparticles and Transdermal Drug Delivery: An Overview. Curr. Pharm. Des..

[38] Salari N., Rasoulpoor S., Valipour E., Mansouri K., Bartina Y., Dokaneheifard S., Mohammadi M., Abam F. (2022). Liposomes, new carriers for delivery of genes and anticancer drugs: A systematic review. Anticancer Drugs.

[39] Akkewar A., Mahajan N., Kharwade R., Gangane P. (2023). Liposomes in the Targeted Gene Therapy of Cancer: A Critical Review. Curr. Drug Deliv..

[40] Kabil M.F., Badary O.A., Bier F., Mousa S.A., El-Sherbiny I.M. (2023). A comprehensive review on lipid nanocarrier systems for cancer treatment: Fabrication, future prospects and clinical trials. J. Liposome Res..

[41] Marques A.C., Costa P.C., Velho S., Amaral M.H. (2023). Lipid Nanoparticles Functionalized with Antibodies for Anticancer Drug Therapy. Pharmaceutics.

[42] Cullis P.R., Hope M.J. (2017). Lipid Nanoparticle Systems for Enabling Gene Therapies. Mol. Ther..

[43] Hou X., Zaks T., Langer R., Dong Y. (2021). Lipid nanoparticles for mRNA delivery. Nat. Rev. Mater..

[44] Schoenmaker L., Witzigmann D., Kulkarni J.A., Verbeke R., Kersten G., Jiskoot W., Crommelin D.J.A. (2021). mRNA-lipid nanoparticle COVID-19 vaccines: Structure and stability. Int. J. Pharm..

[45] Tenchov R., Bird R., Curtze A.E., Zhou Q. (2021). Lipid Nanoparticles─From Liposomes to mRNA Vaccine Delivery, a Landscape of Research Diversity and Advancement. ACS Nano.

[46] Buschmann M.D., Carrasco M.J., Alishetty S., Paige M., Alameh M.G., Weissman D. (2021). Nanomaterial Delivery Systems for mRNA Vaccines. Vaccines.

[47] Zhang H.L. (2023). Current status and patent prospective of lipid nanoparticle for mRNA delivery. Expert Opin. Ther. Pat..

[48] Long J., Yu C., Zhang H., Cao Y., Sang Y., Lu H., Zhang Z., Wang X., Wang H., Song G. (2023). Novel Ionizable Lipid Nanoparticles for SARS-CoV-2 Omicron mRNA Delivery. Adv. Healthc. Mater..

[49] Puccetti M., Schoubben A., Giovagnoli S., Ricci M. (2023). Biodrug Delivery Systems: Do mRNA Lipid Nanoparticles Come of Age?. Int. J. Mol. Sci..

[50] Mendonça M.C.P., Kont A., Kowalski P.S., O’Driscoll C.M. (2023). Design of lipid-based nanoparticles for delivery of therapeutic nucleic acids. Drug Discov. Today.

[51] Swetha K., Kotla N.G., Tunki L., Jayaraj A., Bhargava S.K., Hu H., Bonam S.R., Kurapati R. (2023). Recent Advances in the Lipid Nanoparticle-Mediated Delivery of mRNA Vaccines. Vaccines.

[52] Liu Y., Huang Y., He G., Guo C., Dong J., Wu L. (2024). Development of mRNA Lipid Nanoparticles: Targeting and Therapeutic Aspects. Int. J. Mol. Sci..

[53] Kon E., Ad-El N., Hazan-Halevy I., Stotsky-Oterin L., Peer D. (2023). Targeting cancer with mRNA-lipid nanoparticles: Key considerations and future prospects. Nat. Rev. Clin. Oncol..

[54] Miao L., Zhang Y., Huang L. (2021). MRNA vaccine for cancer immunotherapy. Mol. Cancer.

[55] Feng Q., Zhang Y., Fang Y., Kong X., He Z., Ji J., Yang X., Zhai G. (2023). Research progress of exosomes as drug carriers in cancer and inflammation. J. Drug Target..

[56] Gormley A.J. (2024). Machine learning in drug delivery. J. Control. Release.

[57] Masomi N., Esmaeli E., Ayyoubzadeh S.M., Ghorbani-Bidkorpeh F., Ahmadi M. (2024). Artificial intelligence in nanotechnology for treatment of diseases. J. Drug Target..

[58] Agrahari V., Choonara Y.E., Mosharraf M., Patel S.K., Zhang F. (2024). The Role of Artificial Intelligence and Machine Learning in Accelerating the Discovery and Development of Nanomedicine. Pharm. Res..

[59] Longo J.P.F., de Souza P.E.N., Morais P.C. (2023). Artificial intelligence and machine learning in nanomedicine. What do we expect for 2030?. Nanomedicine.

[60] Akhtar M., Nehal N., Gull A., Parveen R., Khan S., Khan S., Ali J. (2025). Explicating the transformative role of artificial intelligence in designing targeted nanomedicine. Expert Opin. Drug Deliv..

[61] Bannigan P., Aldeghi M., Bao Z., Hsieh F., Macalino S.J., Allen C.J. (2021). Machine learning models to accelerate the design of polymeric long-acting injectables. Nat. Commun..

[62] Kibria M.R., Akbar R.I., Nidadavolu P., Havryliuk O., Lafond S., Azimi S. (2023). Predicting efficacy of drug-carrier nanoparticle designs for cancer treatment: A machine learning-based solution. Sci. Rep..

[63] Wang W., Feng S., Ye Z., Gao H., Lin J., Ouyang D. (2022). Prediction of lipid nanoparticles for mRNA vaccines by the machine learning algorithm. Acta Pharm. Sin. B.

[64] Bae S.H., Choi H., Lee J., Kang M.H., Ahn S.H., Lee Y.S., Choi H., Jo S., Lee Y., Park H.J. (2025). Rational Design of Lipid Nanoparticles for Enhanced mRNA Vaccine Delivery via Machine Learning. Small.

[65] Muneer R., Hashmet M.R., Pourafshary P., Shakeel M. (2023). Unlocking the Power of Artificial Intelligence: Accurate Zeta Potential Prediction Using Machine Learning. Nanomaterials.

[66] Rezvantalab S., Mihandoost S., Rezaiee M. (2024). Machine learning assisted exploration of the influential parameters on the PLGA nanoparticles. Sci. Rep..

[67] Su K., Qiu J., Xu T., Liu S. (2026). Artificial intelligence-guided design of lipid nanoparticles for mRNA delivery. Acta Pharm. Sin. B.

[68] Hoseini B., Jaafari M.R., Golabpour A., Momtazi-Borojeni A.A., Eslami S. (2023). Optimizing nanoliposomal formulations: Assessing factors affecting entrapment efficiency of curcumin-loaded liposomes using machine learning. Int. J. Pharm..

[69] Kashani-Asadi-Jafari F., Aftab A., Ghaemmaghami S. (2022). A machine learning framework for predicting entrapment efficiency in niosomal particles. Int. J. Pharm..

[70] Shirokii N., Din Y., Petrov I., Seregin Y., Sirotenko S., Razlivina J., Serov N., Vinogradov V. (2023). Quantitative Prediction of Inorganic Nanomaterial Cellular Toxicity via Machine Learning. Small.

[71] Mozafari N., Mozafari N., Dehshahri A., Azadi A. (2023). Knowledge Gaps in Generating Cell-Based Drug Delivery Systems and a Possible Meeting with Artificial Intelligence. Mol. Pharm..

[72] Joyce P., Allen C.J., Alonso M.J., Ashford M., Bradbury M.S., Germain M., Kavallaris M., Langer R., Lammers T., Peracchia M.T. (2024). A translational framework to DELIVER nanomedicines to the clinic. Nat. Nanotechnol..

[73] Winkler D.A. (2020). Role of Artificial Intelligence and Machine Learning in Nanosafety. Small.

[74] Ahmad F., Muhmood T. (2024). Clinical translation of nanomedicine with integrated digital medicine and machine learning interventions. Colloids Surf. B Biointerfaces.

[75] Parodi A., Kolesova E.P., Voronina M.V., Frolova A.S., Kostyushev D., Trushina D.B., Akasov R., Pallaeva T., Zamyatnin A.A. (2022). Anticancer Nanotherapeutics in Clinical Trials: The Work behind Clinical Translation of Nanomedicine. Int. J. Mol. Sci..

[76] Csóka I., Ismail R., Jójárt-Laczkovich O., Pallagi E. (2021). Regulatory Considerations, Challenges and Risk-based Approach in Nanomedicine Development. Curr. Med. Chem..

[77] Đorđević S., Gonzalez M.M., Conejos-Sánchez I., Carreira B., Pozzi S., Acúrcio R.C., Satchi-Fainaro R., Florindo H.F., Vicent M.J. (2022). Current hurdles to the translation of nanomedicines from bench to the clinic. Drug Deliv. Transl. Res..

[78] Zhang P., Xiao Y., Sun X., Lin X., Koo S., Yaremenko A.V., Qin D., Kong N., Farokhzad O.C., Tao W. (2023). Cancer nanomedicine toward clinical translation: Obstacles, opportunities, and future prospects. Med.

[79] Tong F., Wang Y., Gao H. (2024). Progress and challenges in the translation of cancer nanomedicines. Curr. Opin. Biotechnol..

[80] D’Mello S.R., Cruz C.N., Chen M.L., Kapoor M., Lee S.L., Tyner K.M. (2017). The evolving landscape of drug products containing nanomaterials in the United States. Nat. Nanotechnol..

[81] Monti S. (2022). Precision Medicine in Radiomics and Radiogenomics. J. Pers. Med..

[82] Hua S., de Matos M.B.C., Metselaar J.M., Storm G. (2018). Current Trends and Challenges in the Clinical Translation of Nanoparticulate Nanomedicines: Pathways for Translational Development and Commercialization. Front Pharmacol..

[83] He H., Liu L., Morin E.E., Liu M., Schwendeman A. (2019). Survey of Clinical Translation of Cancer Nanomedicines-Lessons Learned from Successes and Failures. Acc. Chem. Res..

[84] Shi J., Kantoff P.W., Wooster R., Farokhzad O.C. (2017). Cancer nanomedicine: Progress, challenges and opportunities. Nat. Rev. Cancer.

[85] van der Meel R., Sulheim E., Shi Y., Kiessling F., Mulder W.J.M., Lammers T. (2019). Smart cancer nanomedicine. Nat. Nanotechnol..

[86] Hare J.I., Lammers T., Ashford M.B., Puri S., Storm G., Barry S.T. (2017). Challenges and strategies in anti-cancer nanomedicine development: An industry perspective. Adv. Drug Deliv. Rev..

[87] Ventola C.L. (2012). The nanomedicine revolution: Part 2: Current and future clinical applications. Pharm. Ther..

[88] Arden N.S., Fisher A.C., Tyner K., Yu L.X., Lee S.L., Kopcha M. (2021). Industry 4.0 for pharmaceutical manufacturing: Preparing for the smart factories of the future. Int. J. Pharm..

[89] Shen C., Zhang M., Lu M., Chang E., Gao Z., Ban W., Liu Q., Zuo Z., Jiang C. (2026). Machine learning empowered formulation design, optimization and characterization of nanoparticulate drug delivery systems: Current applications, challenges, and future perspectives. Acta Pharm. Sin. B.

